# Partitioned Gene-Tree Analyses and Gene-Based Topology Testing Help Resolve Incongruence in a Phylogenomic Study of Host-Specialist Bees (Apidae: Eucerinae)

**DOI:** 10.1093/molbev/msaa277

**Published:** 2020-11-12

**Authors:** Felipe V Freitas, Michael G Branstetter, Terry Griswold, Eduardo A B Almeida

**Affiliations:** 1 Laboratório de Biologia Comparada e Abelhas (LBCA), Departamento de Biologia, Faculdade de Filosofia, Ciências e Letras, Universidade de São Paulo, Ribeirão Preto, SP, Brazil; 2 U.S. Department of Agriculture, Agricultural Research Service (USDA-ARS), Pollinating Insects Research Unit, Utah State University, Logan, UT

**Keywords:** Adephaga, Apoidea, ASTRAL, filtering loci, Gene Genealogy Interrogation, Hydradephaga, IQ-TREE, locus partitioning, UCEs

## Abstract

Incongruence among phylogenetic results has become a common occurrence in analyses of genome-scale data sets. Incongruence originates from uncertainty in underlying evolutionary processes (e.g., incomplete lineage sorting) and from difficulties in determining the best analytical approaches for each situation. To overcome these difficulties, more studies are needed that identify incongruences and demonstrate practical ways to confidently resolve them. Here, we present results of a phylogenomic study based on the analysis 197 taxa and 2,526 ultraconserved element (UCE) loci. We investigate evolutionary relationships of Eucerinae, a diverse subfamily of apid bees (relatives of honey bees and bumble bees) with >1,200 species. We sampled representatives of all tribes within the group and >80% of genera, including two mysterious South American genera, *Chilimalopsis* and *Teratognatha*. Initial analysis of the UCE data revealed two conflicting hypotheses for relationships among tribes. To resolve the incongruence, we tested concatenation and species tree approaches and used a variety of additional strategies including locus filtering, partitioned gene-trees searches, and gene-based topological tests. We show that within-locus partitioning improves gene tree and subsequent species-tree estimation, and that this approach, confidently resolves the incongruence observed in our data set. After exploring our proposed analytical strategy on eucerine bees, we validated its efficacy to resolve hard phylogenetic problems by implementing it on a published UCE data set of Adephaga (Insecta: Coleoptera). Our results provide a robust phylogenetic hypothesis for Eucerinae and demonstrate a practical strategy for resolving incongruence in other phylogenomic data sets.

## Introduction

As genome-scale data sets have become more accessible to a broader spectrum of phylogenetic researchers, incongruence among results has become a common occurrence. Incongruence is one of the major challenges faced by researchers using phylogenomic data and there remains little consensus regarding how to easily reconcile differences, especially when conflicting tree topologies receive high statistical support (Jeffroy et al. 2006; Bleidorn and Bleidorn 2017; Stubbs et al. 2020). Moreover, discussion about the underlying causes of incongruence has intensified, with incomplete lineage sorting (ILS) and gene tree estimation error (GTEE) suggested as two of the most likely causes (Edwards 2009; Xi et al. 2015; Arcila et al. 2017; Richards et al. 2018; Betancur-R et al. 2019).

To overcome phylogenetic error due to ILS, approaches using the multispecies coalescent model (MSC) have been developed and shown to perform well in many cases. However, full Bayesian implementations of the model only work with a limited number of taxa or loci due to the computational burden. Phylogenomic data sets for the most part cannot be analyzed with these approaches without subsampling loci and/or taxa (Heled and Drummond 2010; Ogilvie et al. 2017; Flouri et al. 2018). The main alternative to the full implementations of the MSC is “summary” approaches, also called summary methods, in which individual gene trees are estimated separately and all resulting trees are summarized into a single species tree, taking into account gene-tree heterogeneity and the MSC model (Liu et al. 2010; Chifman and Kubatko 2014; Mirarab et al. 2014; Vachaspati and Warnow 2015; Simmons et al. 2016; Zhang, Rabiee, et al. 2018). Summary methods have been shown to perform well when many loci are available and/or when gene trees are accurately estimated (Roch and Warnow 2015; Xi et al. 2015; Mirarab et al. 2016; Nute et al. 2018; Richards et al. 2018).

Two broad strategies have been proposed to address GTEE: the first approach is to infer more accurate gene trees using better programs or models (Chiari et al. 2012; Xi et al. 2015; Mirarab et al. 2016; Van Dam et al. 2017). The alternative strategy is to assess loci based on various parameters, such as proxies of phylogenetic informativeness (e.g., average bootstrap, number of parsimony informative sites), GC content, and/or saturation, and then to remove those loci that are outliers and potentially problematic (Salichos and Rokas 2013; Borowiec et al. 2015; Chen et al. 2015; Pie et al. 2018). These strategies have proven successful at reducing uncertainty in some cases, but confidently resolving incongruence remains a significant challenge, especially when comparing different analytical approaches like concatenation and coalescent-based species tree analyses (Lambert et al. 2015; Arcila et al. 2017; Betancur-R et al. 2019; Gonçalves et al. 2019).

Despite recent improvements in tackling the difficulties related to GTEE, the results are not always satisfactory and new methods and more empirical studies are needed to help determine best practices for phylogenomicists. One of the most promising recent methods developed to resolve incongruences due to GTEE is the Gene Genealogy Interrogation (GGI) approach (Arcila et al. 2017). Under GGI, topological tests are implemented gene-by-gene in a maximum‐likelihood framework allowing for an explicit evaluation of competing topologies of the genealogical history supported with the highest probability by each locus. In one implementation of GGI, two or more constrained gene trees are estimated for single focal nodes according to a predefined set of competing hypotheses. In a modified version of the approach (Arcila et al. 2017; Mirarab 2017), GGI assesses constrained and unconstrained trees in the topological tests, reinforcing the detection of GTEE, because it reduces the chance of stochastic error during the gene-tree search and maximizes phylogenetic signal. If a constrained tree is statistically favored over an unconstrained tree, it indicates stochastic error during the unconstrained gene-tree search (Betancur-R et al. 2019).

In this article, we use a large, diverse group of bees, Eucerinae, to exemplify the methodological challenges of analyzing phylogenomic data and provide guidelines on how to effectively resolve analytical conflict. Eucerinae is a subfamily of Apidae (Hymenoptera: Apoidea), the most emblematic of the seven families recognized in bee classification (Michener 2007). Eucerinae has been recognized as a natural group since the early 1990s, when it was referred to informally as the “eucerine line” (Silveira 1993). In the following decades, the group has been consistently recovered in molecular studies of Apidae based on Sanger-sequencing data (Cardinal et al. 2010; Cardinal and Danforth 2013; Hedtke et al. 2013), and recently with a phylogenomic NGS data set (Bossert et al. 2019). Eucerinae comprises >1,200 species (Ascher and Pickering 2020) in six tribes, and until now had not been the focus of a comprehensive molecular phylogenetic analysis to jointly include all its tribes.

Eucerine bees occur on all continents except Antarctica and Australia, but most of its phylogenetic diversity are concentrated in the New World, especially in the Neotropics, with only 3 of the 57 recognized genera occurring in the Old World (Michener 2007; Moure et al. 2012; Praz and Packer 2014; Dorchin, Danforth, et al. 2018). Elements of Eucerinae have oligolectic (i.e., specialized) associations with specific host plants, suggesting an intriguing evolutionary history of adaptation and specialization that has yet to be studied in detail. Emphorini is largely associated with species of Malvaceae, Convolvulaceae, and Cactaceae (Alves-dos-Santos 1999; Schlindwein 2004; Michener 2007; Schlindwein et al. 2009). The Tapinotaspidini are oil-collecting bees intimately associated with Malpighiaceae and Iridaceae, for oil collecting (Buchmann 1987; Aguiar et al. 2020). Eucerini houses the emblematic squash and gourd bees—*Eucera (Peponapis)* and *E. (Xenoglossa)* associated with *Cucurbita* (Cucurbitaceae) (Hurd et al. 1971), as well as other genera that are apparently oligolectic on other plant groups, for example, *Gaesischia* associated with some Asteraceae (Alves-dos-Santos 1999; Schlindwein 2004), *Santiago mourei* an endemic species from the Cerrado, apparently dependent on pollen from *Vochysia* (Vochysiaceae) (Silveira et al. 2002).

Phylogenetic placement of some eucerine taxa remains unresolved due to either conflicting results among studies, or lack of inclusion in taxon sampling. The latter case is best represented by Teratognathini (*Chilimalopsis* and *Teratognatha*), a rare tribe that has yet to be included in any molecular phylogenetic analysis. Among conflicting results, the most inconsistent finding has been the placement of the genus *Ancyloscelis*, recovered as either the sister group to the remaining Emphorini (Roig-Alsina and Michener 1993; Praz and Packer 2014), or to Exomalopsini (Cardinal et al. 2010; Hedtke et al. 2013; Aguiar et al. 2020). The uniqueness of *Ancyloscelis* justified its recognition as a separate subtribe (Roig-Alsina and Michener 1993), an understanding followed by Aguiar et al. (2020).

Using ultraconserved element (UCE) phylogenomics (Bejerano et al. 2004; Faircloth et al. 2012; Branstetter et al. 2017), we present the most comprehensive phylogenomic data set to date for estimating relationships in Eucerinae. We also conduct multiple analytical strategies, contrasting and combining approaches, including concatenation, coalescence, data filtering, and topological testing using GGI. These strategies have allowed us to produce a comprehensive, well-supported phylogenetic hypothesis for the eucerine bees, to identify areas of topological conflict, and to determine the best ways to resolve these conflicts.

We found that GGI and partitioned gene-tree analyses were particularly helpful at reducing incongruence due to GTEE in our data. To further examine the utility of these two approaches, we reanalyzed a published beetle UCE data set focusing on relationships in the beetle suborder Adephaga (Gustafson et al. 2020). This beetle taxon is traditionally subdivided into two subgroups: the Geadephaga (all terrestrial species—Carabidae and Trachypachidae) and the Hydradephaga (all aquatic species: Amphizoidae, Aspidytidae, Dytiscidae, Gyrinidae, Haliplidae, Hygrobiidae, Meruidae, Noteridae). In recent analyses, the monophyly of Hydradephaga has proven uncertain, despite the attempts to properly resolve it and shed light on the terrestrial–aquatic transitions of adephagan beetles (Maddison et al. 2009; Lawrence et al. 2011; Zhang et al. 2018; Beutel et al. 2020; Gustafson et al. 2020). We address the incongruence among studies and find out that partitioned gene tree searches improves the support for the monophyly of Hydradephaga.

Overall, the examination of the two data sets (bee and beetle) validates the efficiency of our proposed strategy for resolving hard phylogenomic problems and we recommend that this approach be used more often in phylogenomic studies.

## Results

### Taxon Sampling and Matrix Generation

We successfully assembled a data set of 197 species of eucerine bees and related taxa, including all subfamilies of Apidae (see supplementary table S1, Supplementary Material online for the complete taxa list). A total of 148 taxa were sequenced for the first time for UCEs and we captured a total of 2,526 loci for the entire data set. UCE contig coverage for each newly sequenced sample and additional statistics about the data are included in supplementary table S2, Supplementary Material online. Our analyses focus on two sets of loci, filtered for taxon completeness (75p = 75% complete and 90p = 90% complete).

### Phylogenetic Results: Congruence and Conflict

**Fig. 1.**
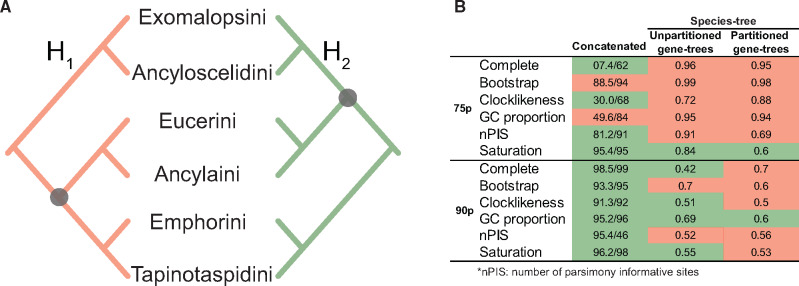
Synthesis of the results obtained with the analyses of the 12 matrices generated for the Eucerinae. (*A*) The two competing hypotheses of the relationship among the three main clades recovered. These were the constraints used to perform GGI approach. (*B*) Hypotheses recovered in all three analyses, with support values for the node highlighted by the black dots in (*A*). Support values in the concatenated analyses are ultrafast bootstrap (UFBoot)/approximate likelihood ratio test (SH-aLRT) and in the summary method analyses are local posterior probability. nPIS, number of parsimony informative sites. Statistics for each data set are shown in supplementary table S3.1, Supplementary Material online.

Both locus sets were analyzed as a concatenated supermatrix using maximum likelihood (ML; IQ-Tree) and using a summary species tree method with gene trees generated under ML and analyzed as single partition. All four results invariably recovered three major clades of Eucerinae with the highest support in all metrics: 1) Eucerini + Ancylaini, 2) Emphorini + Tapinotaspidini, and 3) Exomalopsini + Ancyloscelidini. In these hypotheses, Emphorini is interpreted as monophyletic after the removal of *Ancyloscelis*, which is placed in a much-expanded interpretation of the tribe Ancyloscelidini that also houses *Eremapis*, *Chilimalopsis*, and *Teratognatha*. The sister-group of the clade Eucerini + Ancylaini can either be Emphorini + Tapinotaspidini (henceforth referred to as Hypothesis 1—H1) or Exomalopsini + Ancyloscelidini (Hypothesis 2—H2), as represented in figure 1.

The species tree of the complete 75p matrix recovered H1, whereas the concatenation analyses of both the 75p and 90p matrices and the species tree of the 90p matrix recovered H2 (fig. 1). To explore these incongruences, three different strategies were implemented, as described below.

#### Strategy 1: Filtering Loci

In order to identify best loci or to remove outlier loci, from each of the two matrices, loci were filtered according to five different criteria (1—average bootstrap, 2—clocklikeness, 3—GC proportion, 4—number of parsimony informative sites, and 5—saturation; see Materials and Methods for details). The filtered data sets were analyzed under the same conditions as the complete matrices and the results were similar, with 8 of 20 analyses recovering H1 and 12 analyses recovering H2 (fig. 1).

#### Strategy 2: Partitioned Gene-Tree Analyses

All gene trees were re-estimated with the loci partitioned into core, right flank, and left flank subregions and using a separate substitution model for each subregion. Summary species trees were constructed using the same sets of loci selected for complete matrices and for Strategy 1. With partitioning, ten of our 12 data sets recovered H1, including three (90p-complete, 90p-clocklikeness, and 90p-saturation) that had previously recovered H2 without partitioning. Only two data sets recovered H2 and none of the data sets that previously supported H1 switched to H2 with partitioned gene trees. Additionally, partitioning loci for gene-tree estimation resulted in a significant improvement in the average bootstrap values of the gene trees in the majority of the 32 data sets (supplementary table S4.1, Supplementary Material online).

#### Strategy 3: GGI

**Fig. 2.**
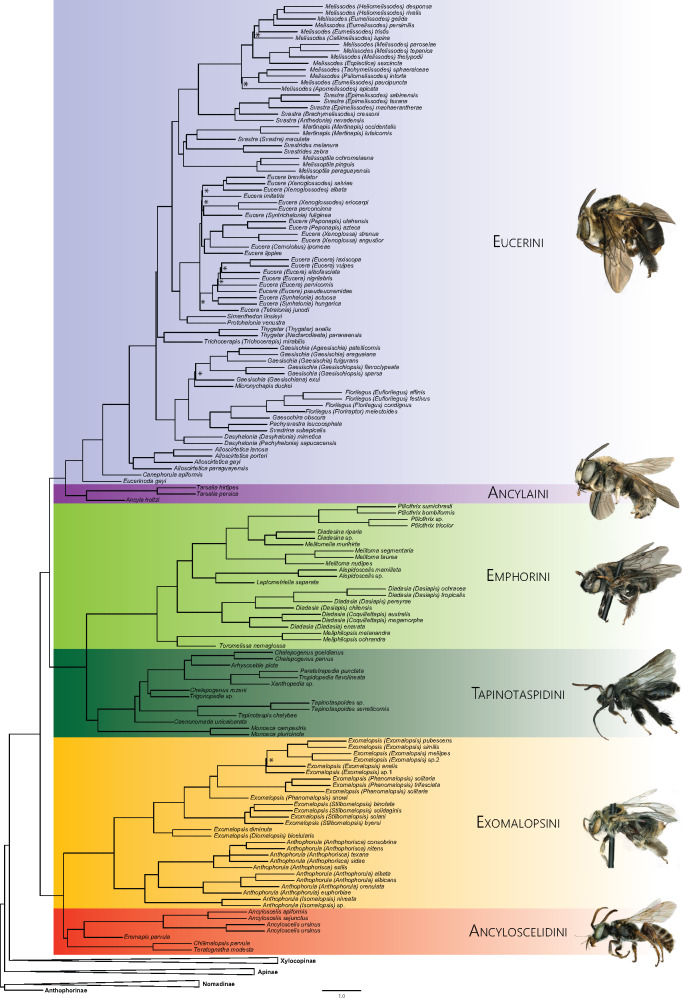
Summary species tree obtained through ASTRAL-MP using all the unconstrained (1,343) and H1 constrained (504) gene trees selected by GGI, from a total of 1,847 gene trees. Branch lengths proportional to coalescent units (scale bar). Nodes with local posterior probabilities <0.95 are indicated by a star (*). Bee photographs (not to scale) from top to button: *Gaesochira obscura* (♀), *Ancyla oranensis* (♂), *Meliphilopsis ochrandra* (♀), *Chalepogenus* sp. (♂), *Exomalopsis (Phanomalopsis) solitaria* (♀), and *Chilimalopsis parvula* (♂).

Taking into account the previous results from strategies 1 and 2 (19 of the 36 results recovered H1, whereas 17 recovered H2—fig. 1), a third strategy was implemented to tackle incongruence among results. We evaluated which predefined genealogical history (H1 or H2) was supported with highest probability by each locus with GGI. We implemented both versions of GGI, the original version that included, for each locus, a gene tree constrained according to both the hypotheses to be tested (i.e., H1 and H2); and the modified version, which statistically compares gene trees from constrained and unconstrained searches. GGI was run using all of the loci present in the 75p locus set. Regardless of the version of GGI used, H1 was supported by many more loci than H2, especially when comparing gene trees with significant *P* values from the AU test (table 1). Following Mirarab’s (2017) suggestion, and based on the results of GGI, we inferred a final, summary species tree using all of the best-constrained gene trees supporting H1 and all of the best-unconstrained gene trees, totaling 1,847 gene trees (gene trees favoring H2 were discarded). The resulting tree (fig. 2) was topologically congruent with other analyses (Supplementary files) with respect to the three main clades discussed above, and it was congruent with H1, with the key node estimated with 100% local posterior probability.

**Table 1.** Summary of the GGI Results of Eucerinae, Showing the Number of Gene Trees Supporting Each Hypothesis.

	Constrained Only		Constrained + Unconstrained		
	H1	H2	H1	H2	Unconstrained
All	1,167 (60.0%)	764 (40.0%)	504 (26.0%)	87 (4.5%)	1,343 (70.0%)
*P* value >0.95	451 (23.0%)	28 (1.5%)	407 (21.0%)	01 (0.1%)	42 (2.0%)

Note.—Percentages are relative to the total number of trees used (1,931), the first line shows all the results and the second only shows results with *P* value >0.95.

### Adephaga Results

**Fig. 3.**
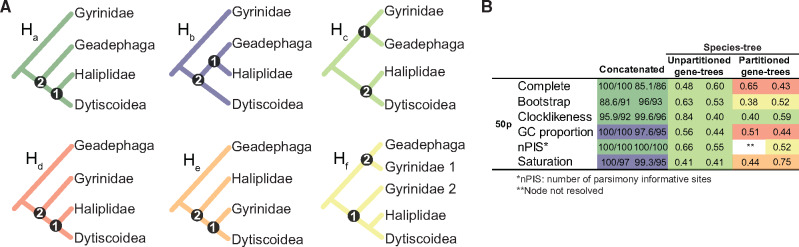
Synthesis of the results obtained with the analyses of the complete 50p matrix of Gustafson et al. (2020) and for the five filtered matrices generated for Adephaga. (*A*) Six competing hypotheses recovered by our analyses of all the six data sets: H_a_–H_f_. Only hypotheses H_d_ and H_e_ represent the monophyly of Hydradephaga, whereas the remaining four hypothesis indicate its paraphyly in relation to Geadephaga. (*B*) Hypotheses recovered in all three analyses, with support values for the nodes highlighted by black dots in (*A*): values on the right refer to node 1 and the left column list values for node 2. Support values in the concatenated analyses are ultrafast bootstrap (UFBoot)/approximate likelihood ratio test (SH-aLRT) and in the summary method analyses are local posterior probability. All four higher taxa represented in this figure were recovered as monophyletic in all analyses except for Gyrinidae in H_f_ (this family was paraphyletic in relation to the remaining taxa when *Spanglerodryrus albiventris*, represented as “Gyrinidae 2,” grouped with Haliplidae, and the remaining gyrinid terminals—“Gyrinidae 1”—grouped with Geadephaga). Statistics for each data set are shown in supplementary table S3.2, Supplementary Material online.

We applied the same procedures used in the eucerine data set to analyze the 50% taxon-complete matrix comprising Adephaga beetles from Gustafson et al. (2020). Using the complete, unfiltered data set, ML recovered the same hypotheses mainly discussed in the original publication (Gustafson et al. 2020: fig. 2)—Hydradephaga paraphyletic, with Gyrinidae sister to Geadephaga + the remaining Hydradephaga (fig. 3*A*: H_a_). Alternatively, summary method analyses yielded the same results as most of their analyses applying this criterion (Gustafson et al. 2020: Supporting Information), with Gyrinidae as sister of Geadephaga, and this clade was placed as the sister group of the remaining Hydradephaga (fig. 3*A*: H_c_). Strategy 1 (filtering loci) yielded the same results using the complete matrix, with varying support values, but a different result was obtained when the loci were filtered by proportion of GC sites and saturation. In the latter analyses, Haliplidae changed position and was recovered as sister to Geadephaga instead of Dytiscoidea (but Hydradephaga was still recovered as paraphyletic in relation to Geadephaga; summarized in fig. 3). When applying Strategy 2 (partitioned gene trees), we recovered four different tree topologies, two being unique to this study. In two of the unique trees, Hydradephaga was recovered as monophyletic (fig. 3: H_d_, H_e_), and in the third tree, Gyrinidae, in addition to Hydradephaga, was recovered as paraphyletic (fig. 3*A*: H_f_). Despite the conflict among species trees, locus partitioning significantly improved the average bootstrap of gene trees (supplementary table S4.1, Supplementary Material online). For Strategy 3 (GGI), we analyzed all six recovered tree topologies (fig. 3*A*: H_a_–H_f_,), and implemented both versions of GGI (including or not unconstrained gene trees) in the topological tests. In contrast to the eucerine bee data set, none of the alternative topologies was favored by GGI as all had fewer than ten loci with significative *P* values (table 2).

**Table 2.** Summary of GGI Results of Adephaga, Showing the Number of Loci Supporting Each Hypothesis.

Constrained Only						
	H_a_	H_b_	H_c_	H_d_	H_e_	H_f_
All	109 (10.1%)	278 (25.9%)	120 (11.2%)	120 (11.2)	236 (22.0%)	213 (19.8%)
*P* value >0.95	2 (0.02%)	9 (0.85%)	1 (0.01%)	1 (0.01%)	9 (0.85%)	1 (0.01%)

Constrained + Unconstrained							
	H_a_	H_b_	H_c_	H_d_	H_e_	H_f_	Unconstrained
All	0	0	0	2 (0.2%)	0	0	1,074 (99.8%)
*P* value >0.95	0	0	0	0	0	0	952 (88.5%)

Note.—Percentages are relative to the total number of trees used (1,076), the first line shows all results and the second only shows results with a *P* value >0.95.

## Discussion

### Incongruence among Concatenation and Species Tree Analyses

Species tree methods, especially as implemented in ASTRAL (Zhang et al. 2018; Yin et al. 2019), the program used here (see Materials and Methods), are more efficient than concatenation at recovering the right tree topology when levels of ILS are high (Davidson et al. 2015; Mirarab and Warnow 2015; Jiang et al. 2020). Assuming H1 is the correct topology of the eucerine bees based on our results and considering that ASTRAL recovered H1 with most of our matrices, whereas H2 was the tree chiefly recovered by concatenation analyses, we can conclude that ILS is an important factor in driving incongruence among results.

However, we must also consider that in the GGI analysis, several constrained gene trees were found to be significantly better than the unconstrained ones, indicating that the unconstrained tree search was unable to find the best tree for those loci. This, in turn, suggests that GTEE could be affecting the performance of the summary species tree methods, which are less effective under high levels of GTEE (Roch and Warnow 2015; Mirarab et al. 2016). The causes of GTEE could be systematic or stochastic (Jeffroy et al. 2006). Systematic errors are the result of model misspecification during tree estimation. When the causes of GTEE are stochastic, phylogenetic informativeness is low, resulting in alternative topologies being equally likely (Jeffroy et al. 2006; Doyle et al. 2015; Richards et al. 2018). The results of the constrained tree search in our implementation of GGI showed that GTEE was most likely a problem for 591 loci (504 supporting H1 and 87 supporting H2) because these loci had a higher likelihood in the constrained tree search as compared with the unconstrained. The GTEE in this case could be explained by absence or reduced phylogenetic informativeness in those loci combined with the fact that constraining the tree-search decreased the number of possible results or reduced the effect of model misspecification.

The tree topology assumed as correct (i.e., H1) was recovered by species tree methods using the complete 75p matrix and both partitioned and unpartitioned gene trees, whereas the 90p matrix only yielded the correct hypothesis when gene trees were estimated after partitioning the individual locus data sets. Partitioning is a strategy that clearly improves gene-tree quality as indicated by their higher bootstrap values (supplementary table S3, Supplementary Material online, and further discussed below). The finding that the 75p locus set was more successful in recovering the H1 result, herein interpreted as the correct one, reinforces previous findings that missing data, per se, is not a problem for summary methods, but the number of gene trees and their quality can in fact muddle phylogenetic conclusions (Nute et al. 2018; Rabiee et al. 2019).

### Partitioning Loci for Gene-Tree Estimation

Partitioning loci for gene-tree inference were employed to incorporate knowledge of sequence heterogeneity within loci in order to improve model fit. This approach has only been attempted once with UCEs (Van Dam et al. 2017), but in a different way. Van Dam et al. (2017) partitioned the flanking regions from one to five partitions according to the length of a given locus, followed by submitting these regions to PartititionFinder2. We used the SWSC-EN method (Tagliacollo and Lanfear 2018) and found this strategy to be a potentially better alternative, given that we observed a significant increase in mean bootstrap support across data sets and the approach is automated in available programs, making it easy to implement. The improved bootstrap support can be explained by the fact that the core and flanking regions of UCEs have different characteristics, with core regions being highly conserved and flanking regions increasing in variability (Faircloth et al. 2012; McCormack et al. 2016). When these regions are treated as different partitions, model fit can be improved.

### Solving the Mysteries of Eucerinae

Our results largely concur with previous conclusions about the systematics of Eucerinae, whether based on Sanger-sequencing data, morphology, or phylogenomic studies with restricted taxon sampling. The main revelation in our phylogenetic result was the novel placement of the phylogenetically unsettled genus *Ancyloscelis*. This taxon was grouped with three other genera that had previously only been studied using morphology: *Chilimalopsis*, *Eremapis*, and *Teratognatha*. We recovered the clade formed by these four genera as sister to Exomalopsini, a result that confirms recent proposals to treat *Ancyloscelis* as separate from other groups (Cardinal et al. 2010; Hedtke et al. 2013; Aguiar et al. 2020) and place this genus together with *Chilimalopsis*, *Eremapis*, and *Teratognatha* in an expanded circumscription of Ancyloscelidini. This result has particular importance for the debate on whether or not to treat *Chilimalopsis* and *Teratognatha* as a separate tribe (Teratognathini, sensu Silveira 1995) or as a subtribe of Exomalopsini (Teratognathina, sensu Michener 2007). Much of the uncertainty on the placement of eucerine groups can be explained by previous limitations in sampling of rare, endemic taxa. In the present study, these limitations were overcome by being able to extract and sequence DNA from museum specimens, made possible by high-throughput DNA sequencers, and especially reduced‐genome data collection methods like sequence capture (Burrell et al. 2015; Blaimer et al. 2016; Derkarabetian et al. 2019).

Beyond the Ancyloscelidini, our results are mostly in agreement with findings from previous phylogenetic studies of eucerine taxa (in particular: Silveira 1995; Silveira and Almeida 2008; Cardinal et al. 2010; Praz and Packer 2014; Dorchin, López-Uribe, et al. 2018; Aguiar et al. 2020). The tribes Emphorini and Exomalopsini, which were never broadly sampled in any previous phylogenetic study using molecular data, had most morphology-based hypotheses confirmed, especially in regard to the monophyly of genera and subgenera (Roig-Alsina and Michener 1993; Silveira 1995; Roig-Alsina 1998). One interesting finding concerning *Exomalopsis* was the position of *E. diminuta*, previously included in the subgenus *E. (Phanomalopsis)* and later removed from this subgenus and hypothesized to be closely related to *E. (Diomalopsis)* (Silveira and Almeida 2008), a conclusion supported by our results.

### Monophyly of Hydradephaga?

The adephagan beetle families traditionally comprising Hydradephaga have been recovered in different phylogenetic positions within Adephaga, depending on the data set and/or the analysis. The results of our analyses highlight the difficulty of this phylogenetic problem, with six different tree topologies recovered with varying placements of Geadephaga, Gyrinidae, Haliplidae, and Dytiscoidea. Two possible explanations for this difficulty were discussed by Gustafson et al. (2020): 1) ancient divergences among groups, with the first splits estimated to have occurred in the early Jurassic (Mckenna et al. 2015; Zhang et al. 2018); and 2) high taxonomic diversity, making a comprehensive taxon sampling hard to implement. The strategy of locus partitioning between core and flanks using SWSC-EN did not yield a single most supported result, but it helped extract phylogenetic signal from the data, which in turn allowed Hydradephaga to be recovered as monophyletic in three analyses (fig. 3*B*: Partitioned gene trees). Importantly, the gene trees based on locus partitioning had a significantly higher average bootstrap in comparison to the unpartitioned trees. In contrast to the more definitive result produced by GGI for the Eucerinae, here the GGI results were indecisive, with roughly the same number of loci supporting each of the six hypotheses. As previously demonstrated by Betancur-R et al. (2019), insufficient taxon sampling can lead to inconclusive results with GGI and we interpret this to be the case in the result of the Adephaga analysis.

## Conclusion

In conclusion, we explored an extensive data set, both in terms of taxonomic breadth and locus sampling, and used it to solve important analytical challenges in the phylogenomic era. We also presented a new approach (locus partitioning between core and flanks using SWSC-EN) to improve the quality of gene trees for summary species tree methods. As a result, we now have a well-supported phylogenetic hypothesis that advances the systematics of Eucerinae and paves the way for future analyses that explore the spatial evolution of these bees and their host-plant associations.

## Materials and Methods

### Taxon Sampling

The sampling for this study included 197 terminal species, 150 representing Eucerinae taxa, and the remaining 47 species are representative of other subfamilies of Apidae (following the classification of Bossert et al. 2019). The sampling of eucerine bees accounted for all six tribes and >80% of the genera recognized in the classification of the subfamily (supplementary table S1, Supplementary Material online). We also included the genera *Chilimalopsis* and *Teratognatha*, suggested to be part of an independent tribe (Teratognathini; Silveira 1995), for the first time in a molecular study. The 47 outgroup species were chosen to include as broad a sample of apid taxa as possible. We sampled representatives of the other four subfamilies, and prioritized taxa with the most UCE loci available. The root of the resulting trees was placed between the clade formed by Anthophorinae and Nomadinae, and the remaining taxa, following the results of Bossert et al. (2019).

### UCE Data Generation

DNA was extracted from one to three legs, depending on the size of the specimen, using the Quick-DNA Miniprep Plus extraction kit (Zymo Research). After extraction, DNA concentration was measured using Qubit 3.0 fluorometer (Thermo Fisher Scientific) and up to 50 ng of input DNA was sheared with a Qsonica Q800R2 to obtain fragments of ∼400–600 bp (30–120 s, 25% amplitude, 10–10 s pulse—the shearing time was calculated according to the age and putative DNA quality of each sample), and the sheared DNA was used as input for NGS library preparation.

For the Illumina library preparation, we used a Kapa Hyper Prep Kit (Kapa Biosystems) and iTru dual-indexing adapters (Glenn et al. 2019). The success of library preparation was assessed by Qubit measurement of DNA concentration and the product of this process was purified with a 1.2× bead cleaning using a substitute for AMPure (Rohland and Reich 2012).

For UCE enrichment, 10–11 samples were pooled at equimolar concentrations and the pool concentration was adjusted to 72 ng/μl using a vacuum centrifuge. The bait set “bee-ant-specific hym-v2,” described in Grab et al. (2019) and based on the UCE loci from Branstetter et al. (2017), was used for enriching the UCE loci. The bait set was synthesized by Arbor Biosciences (formerly MYcroarray). For day one of enrichment, we followed the MYbaits protocol v4.01, and for day two, we followed a more standard UCE protocol available at ultraconserved.org. The custom bait set was diluted 1:4 (1 μl bait, 4 μl H_2_O) and the enrichment incubation was performed at 65 °C for 24 h. After enrichment, the resulting pools were amplified for 17 PCR cycles, purified using SPRI beads, and quantified with Qubit and qPCR (Kapa Library Quantification Kit). The final pool containing all the enriched pools was sequenced at the University of Utah genomics core facility using an Illumina 2500 (PE125, v4 chemistry). A few samples were sent off to Novogene Inc. for Illumina PE150 sequencing.

### Bioinformatics and Matrix Generation

Sequence data were demultiplexed by the sequencing center and then cleaned using Illumiprocessor (Faircloth 2013), a wrapper script that trims adapter contamination and low-quality bases using the trimmomatic package (Bolger et al. 2014). Assembly of sequences into contigs was done using Spades 3.12 (Bankevich et al. 2012), via the PHYLUCE 2.7 (Faircloth 2016) pipeline. The contigs were matched to the UCE probes using PHYLUCE’s program “phyluce_assembly_match_contigs_to_probes” with both min-coverage and min-identity settings set to 80. Extracted UCE contigs were then aligned using MAFFT 7 (Katoh and Standley 2013) and trimmed using trimAl (Capella-Gutiérrez et al. 2009) using default options, both implemented in PHYLUCE.

Two matrices were constructed according to the admissibility of missing data, the first comprised loci sampled for at least 75% of the taxa (75p), whereas the second only included loci with at least 90% of the taxa represented (90p). Statistics for each data set are shown in supplementary table S4, Supplementary Material online.

### Phylogenetic Analyses

Initially partitioned by UCE locus, the two matrices were further partitioned using the Sliding-Window Site Characteristics algorithm—SWSC-EN (Tagliacollo and Lanfear 2018), which uses entropy to separate each UCE locus into core and flanking regions. This strategy makes sense because these loci have an ultraconserved core region surrounded by more variable flanking regions, and partitioning loci this way has been shown to improve model fit (Tagliacollo and Lanfear 2018; Branstetter and Longino 2019). The resulting data subsets were analyzed in PartitionFinder2 (Lanfear et al. 2017) using the rclusterf algorithm. Using IQ-TREE version 1.7-beta17 (Nguyen et al. 2015), we searched for the best substitution models for each of the partitions defined by the SWSC-EN + PartitonFinder2, through ModelFinder (Kalyaanamoorthy et al. 2017). Thereafter, a maximum likelihood analysis, calculating ultrafast bootstrap supports (Minh et al. 2013; Hoang et al. 2018) and SH-like approximate likelihood ratio tests (Guindon et al. 2010) with 1,000 replications each, was performed in the same IQ-TREE session.

Gene trees were estimated in IQ-TREE, searching for the best substitution model for each partition with ModelFinder and calculating ultrafast bootstrap support with 1,000 replications. Summary trees were estimated using ASTRAL-MP (Zhang et al. 2018; Yin et al. 2019) always using default options and calculating Local Posterior Probabilities (Sayyari and Mirarab 2016).

### Phylogenetic Incongruence and Topological Tests

The search for potential sources of incongruence among initial results followed three complementary strategies, detailed below.

#### Strategy 1: Filtering Loci

The statistics for each locus and corresponding gene tree were calculated using AMAS (Borowiec 2016) and a modified version of the script Good Genes (Borowiec et al. 2015—available at https://github.com/marekborowiec/good_genes). After that, for each one of the matrices generated (75p and 90p), we constructed five data sets by selecting 600 best loci according to the following five criteria: 1) highest average bootstrap; 2) clocklikeness, measured here as how the gene tree approximates to an ultrametric tree; 3) lowest GC proportion with respected to AT; 4) highest number of parsimony informative sites (nPIS); and 5) saturation, measured trough regression slope, with higher the values meaning lower saturation potential. The application of these selection criteria resulted in ten filtered data sets (the statistics for each data set are given in supplementary table S4, Supplementary Material online). All these data sets were analyzed with the same parameters described in the previous section.

#### Strategy 2: Partitioned Gene-Tree Analyses

We used the results of SWSC-EN to partition loci into core and flanking regions for gene-tree estimation. The remaining analysis parameters were the same as described before for gene-tree inference. To evaluate if the difference in the mean of mean bootstrap values of all the partitioned gene trees relative to the unpartitioned ones was significant for each data set, they were tested with a nonexact Wilcoxon rank test (Wilcoxon 1945), using the function wilcoxon.test() correcting the *P* values for multiple comparisons using Bonferroni–Holm method (Holm 1979), with the function p.adjust(method = “holm”), both in R (R Core Team 2020).

#### Strategy 3: GGI

We followed the step-by-step protocol made available in the original paper describing the approach (Arcila et al. 2017). A tree search was conducted for each locus by constraining the main clades, in this case, tribes of Eucerinae, to be monophyletic and the relationships among them according to each one of the hypotheses to be tested, but without constraining the relationships within those clades. The resulting trees then had their site likelihood calculated and these values were submitted to a hypothesis test. We applied both versions of the GGI, the first which only includes the constrained trees; and the modified version, which includes the gene trees estimated without any constraint in the hypothesis test (Arcila et al. 2017; Mirarab 2017). The trees were estimated in IQ-TREE, after the search for the best substitution model using ModelFinder (Kalyaanamoorthy et al. 2017); site likelihood scores for each tree were obtained with RAxML (Stamatakis 2014), using the model GTR+GAMMA and ten starting trees (command -N 10). Then, a topological test was conducted for each gene tree by statistically comparing the site likelihood scores of all trees via the approximately unbiased (AU) test in CONSEL v0.1 (Shimodaira 2002). All those unconstrained and H1 constrained gene trees selected by GGI were used to generate a summary tree using ASTRAL-MP with the default parameters.

### Adephaga

For the Adephaga data set, we used all the loci present in the 50p matrix from Gustafson et al. (2020). The alignments used here were the same as those in the original paper, which were kindly provided by the authors. The remaining analytical procedures followed the steps described above for the investigation of the eucerine data set. 

## Supplementary Material

Supplementary data are available at *Molecular Biology and Evolution* online.

## Supplementary Material

msaa277_Supplementary_Datat
